# Quantitative Analysis of Lens Nuclear Density Using Optical Coherence Tomography (OCT) with a Liquid Optics Interface: Correlation between OCT Images and LOCS III Grading

**DOI:** 10.1155/2016/3025413

**Published:** 2016-08-29

**Authors:** You Na Kim, Jin Hyoung Park, Hungwon Tchah

**Affiliations:** ^1^Department of Ophthalmology, University of Ulsan College of Medicine, Asan Medical Center, Seoul, Republic of Korea; ^2^NUNEMISO Eye Center, Seoul, Republic of Korea; ^3^Research Institute for Biomacromolecules, University of Ulsan College of Medicine, Asan Medical Center, Seoul, Republic of Korea

## Abstract

*Purpose*. To quantify whole lens and nuclear lens densities using anterior-segment optical coherence tomography (OCT) with a liquid optics interface and evaluate their correlation with Lens Opacities Classification System III (LOCS III) lens grading and corrected distance visual acuity (BCVA).* Methods*. OCT images of the whole lens and lens nucleus of eyes with age-related nuclear cataract were analyzed using ImageJ software. The lens grade and nuclear density were represented in pixel intensity units (PIU) and correlations between PIU, BCVA, and LOCS III were assessed.* Results*. Forty-seven eyes were analyzed. The mean whole lens and lens nuclear densities were 26.99 ± 5.23 and 19.43 ± 6.15 PIU, respectively. A positive linear correlation was observed between lens opacities (*R* ^2^ = 0.187, *p* < 0.01) and nuclear density (*R* ^2^ = 0.316, *p* < 0.01) obtained from OCT images and LOCS III. Preoperative BCVA and LOCS III were also positively correlated (*R* ^2^ = 0.454, *p* < 0.01).* Conclusions*. Whole lens and lens nuclear densities obtained from OCT correlated with LOCS III. Nuclear density showed a higher positive correlation with LOCS III than whole lens density. OCT with a liquid optics interface is a potential quantitative method for lens grading and can aid in monitoring and managing age-related cataracts.

## 1. Introduction

The Lens Opacities Classification System III (LOCS III) is routinely used in most clinics to quantify cataract density. Since this assessment is based on a slit-lamp evaluation by ophthalmologists, it is limited by the observer bias and reproducibility [1–3]. Although previous studies have attempted quantitative lens grading using lens densitometry, such as the Scheimpflug system [4–6], there were several limitations, including the potential influence of the cornea [7] or the anterior lens surface on the assessment of the internal structure of the lens [8]. Also other studies have pointed out some parameters of Scheimpflug system should be interpretated with caution [2], because the scattering effects of the posterior cortex and posterior capsule are induced by the lens position [9]. Kirkwood et al. described the repeatability and reproducibility of Scheimpflug imaging for lens densitometry in eyes with and without cataract. Although the magnitude of the density was higher in the cataract group, the cataract group displayed marginally less repeatability than did the noncataract group [10]. CATALYS^®^ precision laser system is a newly developed equipment for femtosecond laser-assisted cataract surgery, and this system comprises a high quality spectral domain OCT using a liquid optics interface (<11 *μ*m depth resolution, >12 mm image depth). Conventional time-domain anterior segment OCT can only capture either the anterior or the posterior half of the lens at each time and integrate two disjointed images into a whole image [11]. In contrast to the time-domain OCT, this spectral OCT system can visualize entire lens anatomy, including the posterior capsules, and can be used to measure cataract density objectively [12, 13]. To the best of our knowledge, commercialized technical equipment that can measure posterior capsule and the objective lens densities for the assessment of age-related nuclear cataract is not available at this time. Therefore, the aim of the current study is to quantify lens density using OCT with a liquid optics interface and to evaluate the correlation between lens density (obtained using OCT images), BCVA, and LOCS III lens opalescence grading score system.

## 2. Materials and Methods

### 2.1. Patients

The medical records of patients who underwent femtosecond laser-assisted cataract surgery at the Asan Medical Center (Seoul, Republic of Korea) between December 2013 and June 2015 were reviewed. Patients aged ≥60 years and with an LOCS III lens opalescence grading system score >3 were included in the study. Exclusion criteria included any ocular pathology that could influence quantitative OCT imaging and any other specified type of cataract. This study was approved by the institutional review board of the Asan Medical Center (Seoul, Republic of Korea) (IRB no. 2016-0645) and adhered to the tenets of the Declaration of Helsinki.

### 2.2. Patient Assessment

Baseline examinations included measurement of uncorrected distance visual acuity (UDVA) and corrected distance visual acuity (BCVA), manifest refraction using the fogging technique, slit-lamp examination of the anterior segment, and dilated fundoscopy. Intraocular pressure (IOP) was measured using a noncontact tonometer (Canon TX-20, Canon, NY, USA). Other measurements included specular microscopy (CELLCHECK SL, Konan medical, USA), optical biometry (IOLMaster, Zeiss, Germany), slit scanning corneal tomography (ORBSCAN, BAUSCH & LOMB, USA), and macular and optic disc optical coherence tomography (OCTIII, Heidelberg Engineering, Germany). All baseline examinations were performed by specially trained optometrists. Prior to the surgery, the purpose, as well as possible complications, was explained to each patient, after which an informed written consent was obtained.

### 2.3. Lens Density Quantification

Using a traditional method, cataract density was measured based on LOCS III grading score following a slit lamp examination under mydriasis with tropicamide 0.5% and phenylephrine HCl 0.5%. To control observer bias, only one specially trained ophthalmologist (Hungwon Tchah) performed every slit lamp examination. The LOCS III grading score was assigned a numeric scale to reflect the degree of nuclear opacity (3.0–6.0) in increments of 0.50 and the eyes with a score >6.0 were excluded due to the limited resolution of posterior capsule in OCT images.

**Figure 1:**
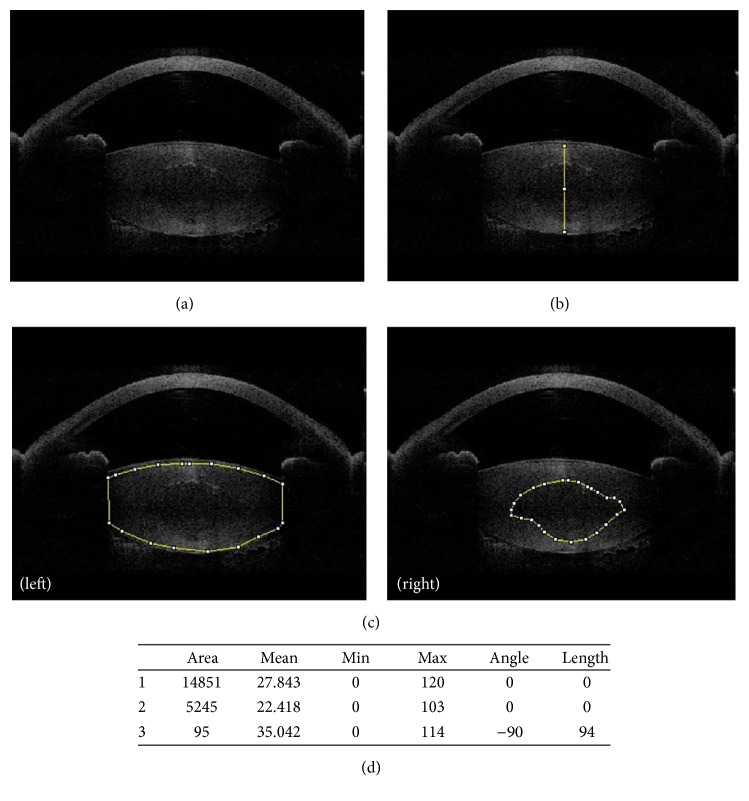
The procedure for measuring lens opacity, lens nuclear density, and lens thickness using ImageJ program. (a) A CATALYS OCT image of anterior segment including entire lens. (b) The procedure for measuring lens thickness from the image. (c) The procedure for measuring whole lens density (left) and nuclear density (right). (d) Lens density and lens thickness values measured using ImageJ (pixel intensity unit).

In this study, the OCT images from CATALYS precision laser system were acquired on the day of cataract surgery and femtosecond laser-assisted nuclear phacoemulsification was performed in all cases. Although OCT images offer sagittal and axial views simultaneously, only sagittal view images were analyzed to increase correlation with slit lamp examinations. Using the ImageJ program, the contours of the entire lens, nucleus, and lens thickness were measured manually by one independent observer (You Na Kim). The mean density was calculated automatically and scored in the range 0–50 in pixel intensity units (PIU) (Figure 1).

### 2.4. Statistical Analysis

To identify the correlations between LOCS III, BCVA, and nuclear density from OCT images, Spearman correlation coefficient analysis was carried out using SPSS software (version 21, IBM, USA) and a *p* < 0.05 was considered statistically significant.

## 3. Results

### 3.1. Patient Characteristics

A total of 47 eyes from 47 patients were included in the study (23 male, 24 female) and their mean age was 73.13 ± 6.35 years (range 61–87). The mean preoperative BCVA was 0.61 ± 0.78 logMAR units and the mean spherical equivalent was −0.72 ± 2.55 diopters. The mean nuclear opalescence obtained from LOCS III was 4.41 ± 1.03 (range 3.00–6.00). From OCT images analysis, the mean whole lens and nuclear densities were found to be 26.99 ± 5.23 and 19.43 ± 6.15 PIU, respectively (Table 1). Among 47 eyes, subgroups of nuclear, cortical, and posterior subcapsular cataract were analyzed by their age, preoperative BCVA, and LOCS III. Comparing three cataract groups with Kruskall Wallis test, only LOCS III grade showed significant difference between each cataract group (*p* = 0.000, Table 2).

### 3.2. Lens Density and Inter Measurement Correlation

**Figure 2:**
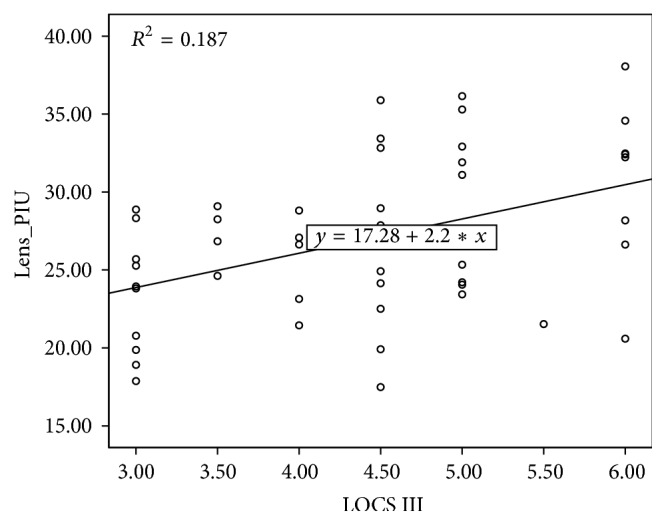
The relationship between whole lens opacity density measured using optical coherence tomography (OCT) and LOCS III.

**Figure 3:**
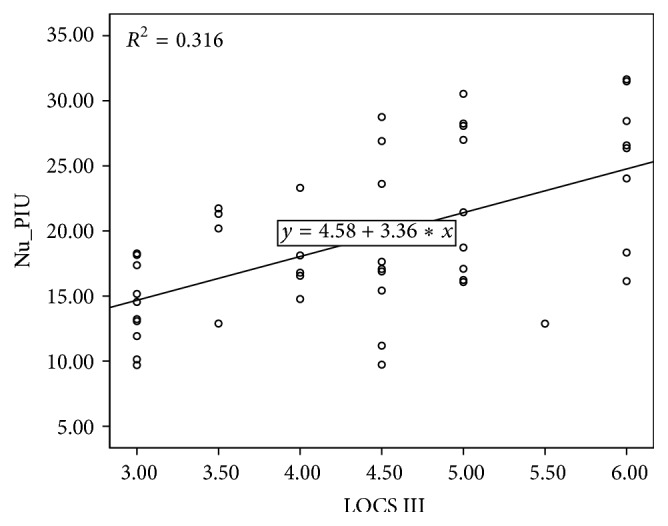
The relationship between lens nuclear densities measured using optical coherence tomography (OCT) and LOCS III.

The lens opacity, measured using OCT, positively correlated with LOCS III score (*R* ^2^ = 0.187, *p* < 0.01) (Figure 2). The nuclear density showed a higher positive correlation (*R* ^2^ = 0.316, *p* < 0.01) (Figure 3). The analysis of lens thickness and preoperative SE revealed no specific correlation between them (*R* ^2^ = 0.064, *p* < 0.05). In addition, the relationship between preoperative BCVA and LOCS III and OCT-measured grading was compared and positive correlation was observed only with LOCS III score (*R* ^2^ = 0.264, *p* < 0.01).

## 4. Discussion

Though age-related cataract is one of the major causes of blindness worldwide, the absence of an objective grading system continues to be a challenge [14]. Phacoemulsification has been used as a definite treatment modality for age-related cataract. However, its irreversibility draws our attention while making an objective grading of cataract density and in predicting phacodynamics during cataract surgery. Several clinical classifications, including LOCS III, have been used to quantify lens density. However, their use has been limited due to a lack of reproducibility and intraobserver variations [2, 3]. In this respect, there have been numerous trials to standardize cataract grading systems. With the development of camera based lens densitometry, such as Scheimpflug camera, there were trials evaluating the correlation between lens image density and cataract opalescence [15]. Cabot et al. described that the parameters, Objective Scatter Index (OSI) and Modulation Transfer Function (MTF), from Optical Quality Analysis System (Visiometrics, Terrasa, Spain) are correlated with the severity of the cataract according to the LOCS III grade and with visual acuity in nuclear, cortical, posterior subcapsular cataract subgroups. In consequence of these results, the study determined that OSI and MTF could be useful parameters to discriminate objective cataract grading of crystalline lens [16]. In addition, ocular wavefront aberration was applied from ray tracing system to evaluate visual quality distortion related to nuclear sclerosis [17, 18]. Although these are developed as reproducible methods for cataract grading, Scheimpflug camera imaging was limited by the use of a single peak value of lens density to compare the average nuclear densities [19, 20] and it cannot visualize posterior cortex and capsule appropriately. In addition, OSI or wavefront aberration analysis cannot provide direct information of the lens opacification but only reflects information affected by scattering [21, 22]. Contrary to the methods mentioned above, anterior segment OCT can reveal the complete lens anatomy directly. In the recent study directed by Skiadaresi et al., images of anterior and posterior segment from spectral or Fourier-domain OCT have proven to be very successful and to get constant result regardless of its lens magnification [3]. Furthermore, newly developed OCT with a liquid optics interface can visualize posterior capsule and facilitates the analysis of lens density with greater objectivity. In this study, the correlations between LOCS III and lens density, obtained from OCT with liquid optics interface imaging, and BCVA were evaluated. Particularly, lens nuclear density and the LOCS III grading revealed a higher positive correlation than whole lens density (*R* ^2^ = 0.316, *R* ^2^ = 0.187, *p* < 0.01, resp.). It seems that it reflects that nuclear opalescence of LOCS III grading was classified as the color of nucleus of cataract. To our knowledge, this is the first study, which confirms the correlation between LOCS III and AS OCT images, acquired using a liquid optics interface, including entire posterior capsule of lens as a visual pathway.

Although OCT with liquid optics interface imaging can image the entire lens, as opposed to other anterior-segment OCT techniques, it has several limitations associated with the evaluation of lens anatomy [12, 13, 23]. Even when pupils were fully dilated, the posterior shadowing of pupils concealed the margin of the lens cortex. This imposed a restriction on identifying the margins covering the entire lens capsule and could be the reason behind the observed higher correlation between lens nuclear density and LOCS III score compared to whole lens opacity. Since a thick nucleus inhibits media penetration, the posterior cortex and posterior capsule linings were hardly visible in a lens densitometry system, in advanced cataract [23]. In addition, other types of cataract, except age-related cataract, could not be evaluated in this study, which is one of the limitations. Apart from these technical limitations, the small number of cases is another limitation of this study to verify its repeatability and reproducibility. In this respect, further study should be designed with bigger sample size to make up for the limitation of reproducibility in this study.

Concerning quantifying cataract grading, OCT-based measurement of lens density is more objective and reproducible compared with LOCS III grading. Therefore, these results imply that the lens densitometry using OCT with liquid optics interface imaging can be one of the options for cataract grading. Like OCT with liquid optics interface imaging, the advent of objective cataract grading imaging tools enables the evaluation of cataract progression in long-term follow-up patients and providing consistent medical consultation [15, 24, 25].

## 5. Conclusion

The development of anterior-segment imaging modalities enables the visualization of lens density for cataract surgery [26]. Among these modalities, CATALYS precision laser system providing OCT images can capture an image of the entire lens at one go [12, 13]. In conclusion, PIU of whole lens and nuclear densities obtained from OCT with a liquid optics interface correlated with LOCS III score and BCVA. The nuclear density showed a higher positive correlation with LOCS III than the whole lens density and BCVA. In other words, CATALYS OCT-based measurement of lens density was analyzed to establish an objective cataract grading system and the correlation with LOCS III was confirmed. Although this method of cataract grading system has several limitations to facilitate its use as a supplement to LOCS III, this study shows the possibility of a novel lens density measurement for cataract diagnosis [27]. In addition, it can be helpful in making consistent and objective decisions during the management of cataract.

## Figures and Tables

**Table 1:** Patient demographic data.

Parameter	Range	Mean ± SD
Eyes (*n*)		47
Patients (*n*)		47
Sex (male : female)		23 : 24
Age (years)	61.00–87.00	73.13 ± 6.35
LOCS III grade	3.00–6.00	4.41 ± 1.03
Preop. BCVA (logMAR)	0.00–3.00	0.61 ± 0.78
Preop. SE (diopters)	−11.00–+3.13	−0.72 ± 2.55
Whole lens density (PIU)	17.49–38.06	26.99 ± 5.23
Lens nuclear density (PIU)	9.69–31.65	19.43 ± 6.15
Lens thickness (pixels)	90.00–163.00	110.32 ± 11.95

**Table 2:** Demographic data and Lens Opacities Classification System (LOCS) III cataract grading.

Parameter	Cataract			
	Nuclear	Cortical	PSC	*p* value
Eyes (*n*)	24	12	14	
Age (years)	72.96 ± 6.75	73.75 ± 6.45	72.36 ± 5.96	0.800
Preop. BCVA (logMAR)	0.69 ± 0.90	0.31 ± 0.27	0.69 ± 0.78	0.322
LOCS III grade	4.85 ± 0.99	3.50 ± 0.64	4.32 ± 0.82	0.000^*∗*^

Statistical significance between the three study groups analyzed by Kruskall Wallis test (*∗*: *p* < 0.05).
